# ATF6 prevents DNA damage and cell death in colon cancer cells undergoing ER stress

**DOI:** 10.1038/s41420-022-01085-3

**Published:** 2022-06-25

**Authors:** Rossella Benedetti, Maria Anele Romeo, Andrea Arena, Maria Saveria Gilardini Montani, Livia Di Renzo, Gabriella D’Orazi, Mara Cirone

**Affiliations:** 1 Department of Experimental Medicine, Sapienza University of Rome, Viale Regina Elena 324, 00161 Rome, Italy; 2 Laboratory Affiliated to Istituto Pasteur Italia-Fondazione Cenci Bolognetti, Viale Regina Elena 291, 00161 Rome, Italy; 3 Department of Neurosciences, Imaging and Clinical Sciences, University G. D’Annunzio, Via dei Vestini 33, 66100 Chieti, Italy; 4 Department of Research and Technological Innovation, IRCCS Regina Elena National Cancer Institute, Via Elio Chianesi 53, 00128 Rome, Italy

**Keywords:** Cancer therapy, Cell death

## Abstract

Colon cancer represents one of the most common and aggressive cancers in its advanced state. Among the most innovative anti-cancer approaches, the manipulation of UPR is a promising one, effective also against cancers carrying dysfunctional p53. Interestingly, it is emerging that UPR cross-talks with DDR and that targeting the interplay between these two adaptive responses may be exploited to overcome the resistance to the single DDR- and UPR-targeting treatments. Previous studies have highlighted the role of IRE1 alpha and PERK UPR sensors on DDR, while the impact of ATF6 on this process remains under-investigated. This study shows for the first time that ATF6 sustains the expression level of BRCA-1 and protects colon cancer cells from the cytotoxic effect of ER stressors DPE and Thapsigargin. At molecular level, ATF6 activates mTOR to sustain the expression of HSP90, of which BRCA-1 is a client protein. Therefore, pharmacological or genetic inhibition of ATF6 promoted BRCA-1 degradation and increased DNA damage and cell death, particularly in combination with Adriamycin. All together this study suggests that targeting ATF6 may not only potentiate the cytotoxic effect of drugs triggering ER stress but may render colon cancer cells more sensitive to Adriamycin and possibly to other DNA damaging agents used to treat colon cancer.

## Introduction

Cancer cells are exposed to a variety of intrinsic and extrinsic stress and therefore activate several adaptive responses to keep surviving. Among those, one of the most important is the unfolded protein response (UPR), activated by conditions that induce endoplasmic reticulum (ER) stress, such as the accumulation of misfolded proteins into the ER or oxidative stress [1]. UPR is orchestrated by the signaling initiated by the three independents, although interconnected, sensors, namely Inositol-requiring enzyme 1 (IRE1) alpha, PKR-like endoplasmic reticulum kinase (PERK), and activating transcription factor 6 (ATF6). Interestingly, UPR cross-talks with other adaptive responses, playing a key role in the survival of cancer cells, such as autophagy [2, 3] or DNA Damage Response (DDR) [4]. The latter is essential for repairing DNA damage that frequently occurs in cancer cells, due to the high replication rate and exposure to hypoxia, condition that can itself impair the DNA repair pathways [5]. Among the UPR sensors, it has been reported that the spliced transcription factor X-box binding protein 1 (XBP1s), generated from the splicing of XBP1 mediated by IRE1 alpha endoribonuclease activity, plays a key role in protecting cells during ER stress [6]. Interestingly, XBP1s also sustains the expression level of several molecules involved in DNA homologous repair (HR) such as BRCA-1 and RAD51 and non*-*homologous end-joining (NHEJ) repair such as XRCC1 [4]. Regarding PERK UPR arm, it is known that it may play a pro-survival role during ER stress by blocking protein translation, by triggering autophagy to help cells to ride out of unwanted materials and damaged organelles, and by activating NF-E2-related factor 2 (NRF2), to reduce oxidative stress [7]. However, when the protective functions of UPR are overwhelmed, PERK/eIF2 alpha axis activation can also promote cell death, i.e., by upregulating the pro-apoptotic molecule C/EBP Homologous Protein (CHOP), that alters the balance between the pro-apoptotic and anti-apoptotic Bcl-2 family proteins [8]. Regarding the impact of PERK/eIF2 alpha axis on DDR, it has been reported that its activation may be involved in the proteasomal degradation of RAD51, increasing DNA damage [9, 10]. Finally, few studies have investigated whether ATF6 might influence DDR, while it is known that it mainly sustains cell survival in the course of ER stress, for example by upregulating the ER chaperone Binding immunoglobulin protein (BiP), that promotes protein re-folding and lowers ER stress [11]. We have previously shown that the inhibition of ATF6 by ceapinA7 exacerbated ER stress induced by 3,4-dihydroxyphenyl ethanol (DPE), an antioxidant compound contained in the olive oil, increasing its cytotoxic effect against colon cancer cells. ATF6 inhibition was more effective than IRE1 alpha and PERK inhibition in potentiating DPE-induced cell death and was the only arm, whose inhibition potentiated DPE-induced cell death in colon cancer cells carrying mutant p53 (mutp53). In these cells, DPE/ceapinA7 combination downregulated mutp53 expression level, while in colon cancer cells carrying wtp53, it induced the activation of p53 [12]. Based on the latter finding and considering that DNA damage is one of the most important triggers of wtp53 activation, in this study we investigated this aspect and evaluated the occurrence of DNA damage in colon cancer cells in which ATF6 was inhibited during the treatments by DPE or Thapsigargin, that is one of the most characterized ER stressors. We correlated DNA damage with the expression of one of the most important players of DNA damage HR, BRCA-1. As ATF6 has been reported to sustain mammalian target of rapamycin (mTOR) activation in the course of ER stress [13] and considering that this pathway, among other numerous functions [14] may control heath shock protein (HSP) expression [15], we then investigated whether ATF6 inhibition could affect HSP90 expression and impair the stability of BRCA-1, known to be a HSP90 client protein.

## Results

### ATF6 sustains cell survival by preventing DNA damage and BRCA-1 downregulation in colon cancer stressed by DPE treatment

**Fig. 1:**
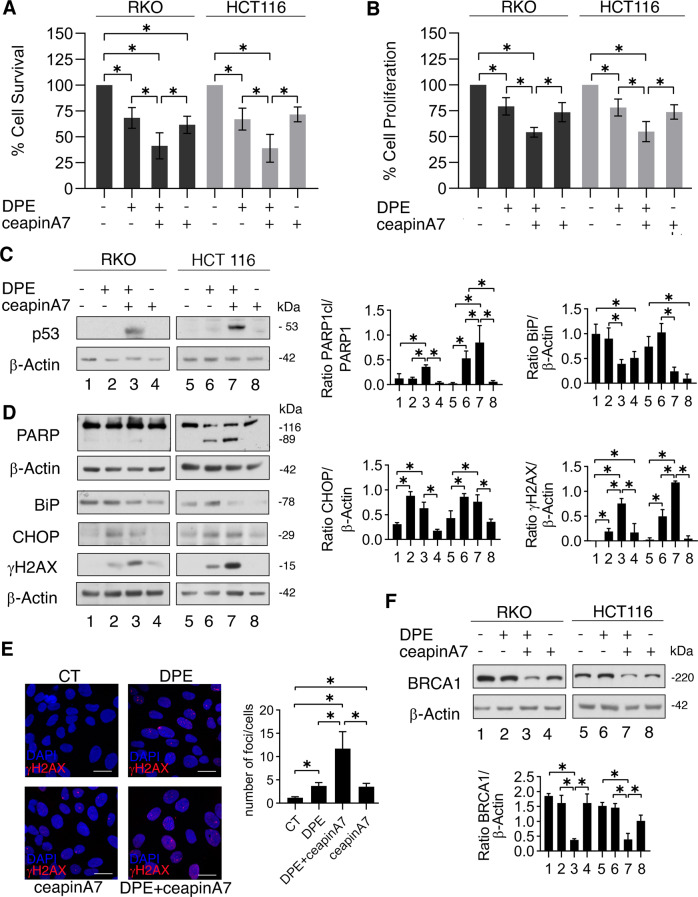
ATF6 inhibition reduces survival in DPE-treated cells and increases DNA damage which correlates with BRCA-1 reduction. RKO and HCT116 cells were pre-treated or not with ceapinA7 (12 μM) for 1 h and then treated or not with DPE (100 μM) or left untreated, as control. After 48 h of treatments, **A** cell viability was measured by a Trypan Blue exclusion assay, the histograms represent the mean ± S.D. of live cells as percent of untreated control cells. By using the KERN index (R), we found that the combination of DPE plus ceapinA7 induced a synergistic cytotoxic effect in both RKO and HCT116 cells (R > 1). **B** Cell proliferation was measured by MTT assay, the histograms represent the mean ± S.D. of the ratio between the OD of treated cells and control cells. *p* value: *<0.05. **C**, **D** Protein expression level of p53, PARP cleavage, BiP, CHOP, γH2AX was evaluated by western blot analysis. β-Actin was used as loading control and one representative experiment is shown. The histograms represent the densitometric analysis of the ratio of specific protein and the appropriate control of three different experiments. Data are represented as the mean plus S.D. *p* value: *<0.05. **E** γH2AX foci (red) were assessed by IFA in RKO cell line. DAPI (blue) was used for nuclear staining. One representative experiment out of three is reported. The histograms represent the mean plus S.D. of the number of foci/cell from three different experiments. Bars = 20 μm, *p* value: *<0.05. **F** Western blot analysis of BRCA-1 expression level. β-Actin was used as loading control and one representative experiment is shown. The histograms represent the densitometric analysis of the ratio of BRCA-1/β-Actin of three different experiments. Data are represented as the mean plus S.D. *p* value: *<0.05.

We first confirmed that ATF6 inhibition by ceapinA7 could further reduced cell survival in DPE-treated RKO colon cancer cells, as previously reported [12]. We then extended this study to another wtp53-carrying colon cancer cell line, HCT116, obtaining similar results either in a Trypan Blue (Fig. 1A) and in a MTT assay (Fig. 1B). As previously observed in RKO cells, we found that DPE/ceapinA7 combination triggered p53 activation also in HCT116 cells (Fig. 1C). We then found that PARP cleavage increased in RKO and HCT116 cells treated with DPE in combination with ceapinA7 (Fig. 1D), according to the reduction of cell survival. Given that p53 is mainly activated in response to DNA damage, we next evaluated the phosphorylation of H2AX (γH2AX), a marker of DNA damage, in cells undergoing such combination treatment. As shown in Fig. 1D, γH2AX expression level was higher in cells treated by DPE/ceapinA7 compared to those treated by DPE or ceapinA7 alone, both in RKO and HCT116 cell lines. The efficacy of ATF6 inhibition by ceapinA7 was evaluated by assessing the reduction of BiP, whose expression is known to be strongly influenced by ATF6 activity [16]. We also observed an upregulation of the pro-apoptotic UPR molecule CHOP in stressed cells, although its expression level did not further increase with DPE/ceapinA7 in comparison to the single DPE treatment (Fig. 1D), given that ATF6 contributes to CHOP upregulation [17].

The occurrence of DNA damage was further investigated by IFA, that showed that the number of γH2AX-positive foci induced by DPE further increased in combination with ceapinA7 (Fig. 1E). We then asked whether the stronger DNA damage induced by DPE/ceapinA7-treatment could be due to an impairment of DDR in colon cancer cells. Therefore, we evaluated the expression level of BRCA-1, a key molecule of HR pathway, and found that it was downregulated by DPE/ceapinA7 combination compared to the single DPE or ceapinA7 treatments, both in RKO and HCT116 cell lines (Fig. 1F). These results suggest that ATF6 played a role in preventing BRCA-1 downregulation and DNA damage in colon cancer cells undergoing DPE treatment.

### ATF6 protects from DNA damage and cell death colon cancer cells treated by Thapsigargin

**Fig. 2:**
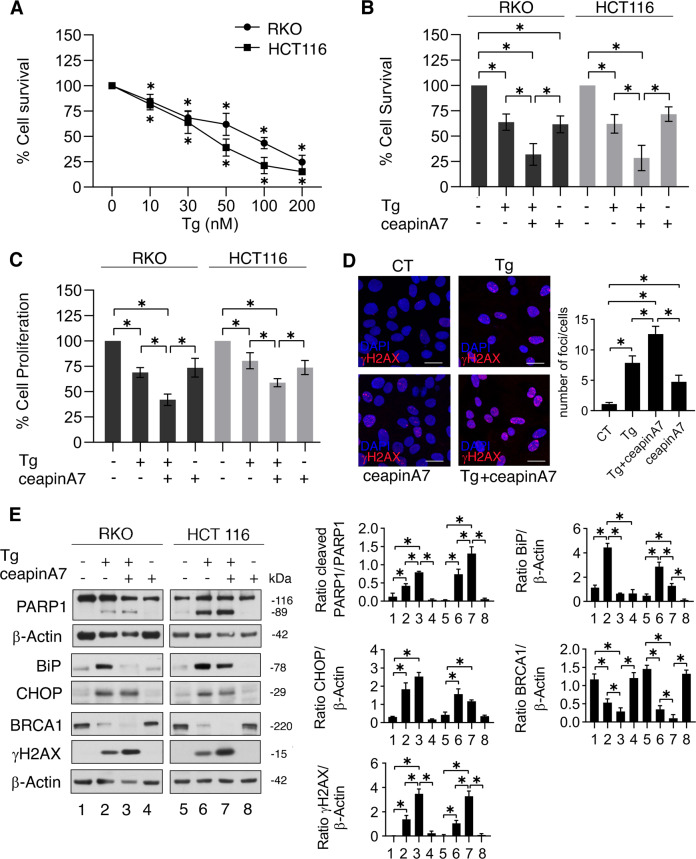
ATF6 inhibition reduces survival, increases DNA damage, and reduces BRCA-1, although in Thapsigargin-stressed cells. **A** RKO and HCT116 cell lines were treated different doses of Thapsigargin (Tg) (10, 30, 50, 100, and 200 nM) or left untreated, as control. Cell viability was measured by a Trypan Blue exclusion assay after 48 h of culture, the dots represent the mean ± S.D. of live cells as percent of untreated control cells. *p* value: *<0.05. RKO and HCT116 cells were pre-treated or not with ceapinA7 (12 μM) for 1 h and then treated or not with Thapsigargin (30 nM) or left untreated, as control. After 48 h of treatments, **B** cell viability was measured by a Trypan Blue exclusion assay, the histograms represent the mean ± S.D. of live cells as percent of untreated control cells. By using the KERN index (R), we found that the combination of Thapigargin and ceapinA7 induced a synergistic cytotoxic effect in both RKO and HCT116 cells (R > 1). **C** Cell proliferation was measured by MTT assay, the histograms represent the mean ± S.D. of the ratio between the OD of treated cells and control cells. *p* value: *<0.05. **D** γH2AX foci (red) were assessed by IFA in RKO cell line. DAPI (blue) was used for nuclear staining. One representative experiment out of three is reported. The histograms represent the mean plus S.D. of the number of foci/cell from three different experiments. Bars = 20 μm. *p* value: *<0.05. **E** Protein expression level of PARP cleavage, BiP, CHOP, BRCA-1, and γH2AX was evaluated by western blot analysis. β-Actin was used as loading control and one representative experiment is shown. The histograms represent the densitometric analysis of the ratio of specific protein and the appropriate control of three different experiments. Data are represented as the mean plus S.D. *p* value: *<0.05.

To confirm the role of ATF6 in sustaining BRCA-1 expression level and DNA repair in the course of ER stress, we used another well-characterized ER stressor, Thapsigargin. As shown in Fig. 2A, this treatment induced a dose-dependent cytotoxic effect in colon cancer cells and, as for DPE, Thapsigargin-mediated reduction of cell survival (Fig. 2B) as well as the impairment of cell proliferation (Fig. 2C) increased in combination with ceapinA7. Accordingly, Thapsigargin plus ceapinA7 enhanced the number of γH2AX-positive foci in comparison to the single treatments (Fig. 2D). Furthermore, Thapsigargin/ceapinA7 induced a more intense cleavage of PARP and increased γH2AX expression level (Fig. 2E). Also in this case, we demonstrated that ATF6 inhibition downregulated BiP and that CHOP was upregulated in stressed cells (Fig. 2E). As for DPE/ceapinA7-treatment, BRCA-1 expression level was reduced also by Thapsigargin/ceapinA7 (Fig. 2E). However, we observed that Thapsigargin, at the dose used in this experiment (30 nM), was per se able to downregulate BRCA-1 and to induce a mild DNA damage, although both effects were strengthened by its combination with ceapinA7 (Fig. 2D, E).

### Thapsigargin at the higher dose reduces BRCA-1 mRNA expression while ceapinA7 does not influence it

**Fig. 3:**
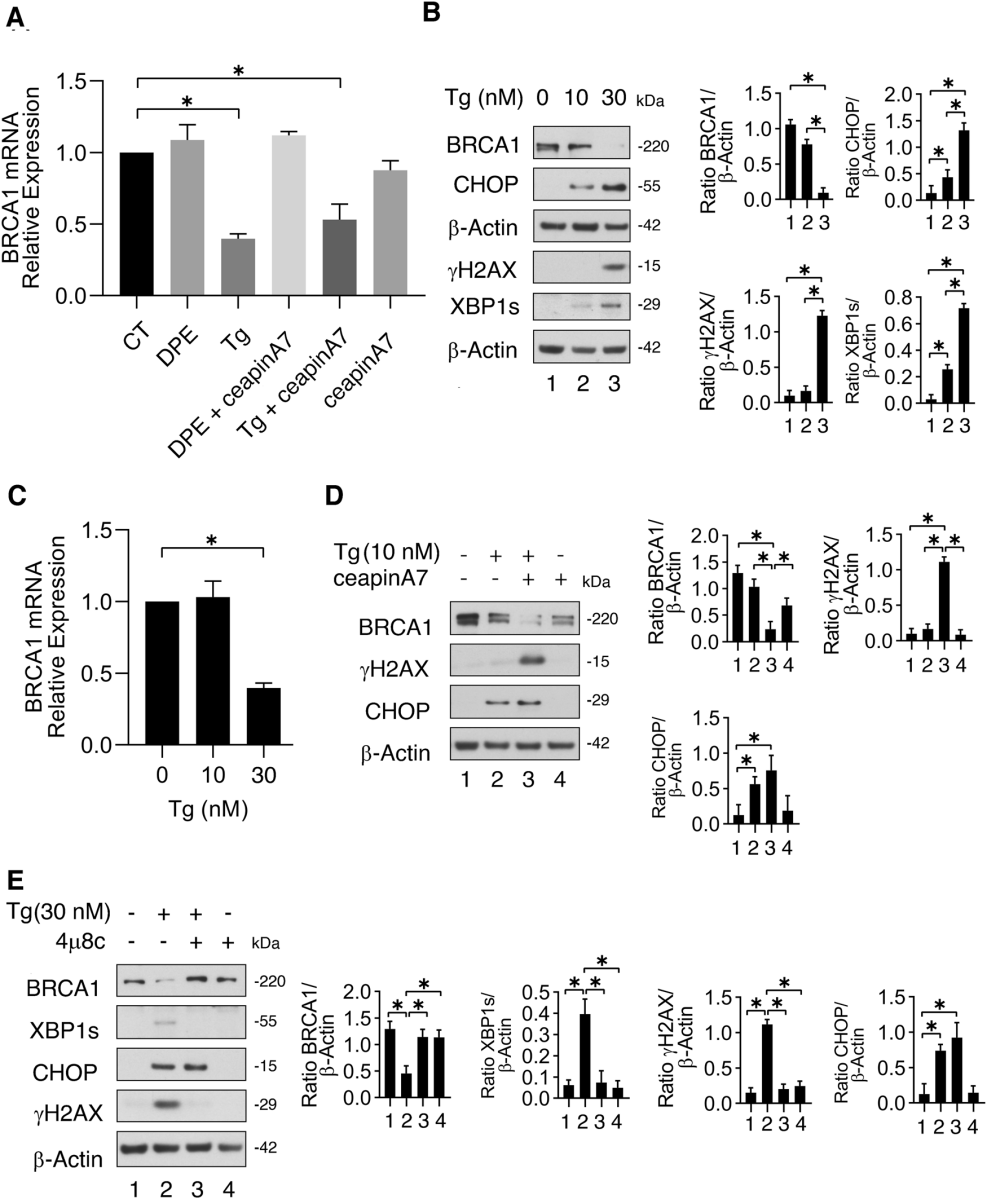
ATF6 inhibitor downregulation of BRCA-1 occurs at post-transcriptional level, while Thapsigargin reduces BRCA-1 mRNA in a dose-dependent manner. **A** qRT-PCR of BRCA-1 in RKO cell line pre-treated or not with ceapinA7 (12 μM) and treated or not with DPE (100 μM) or Thapsigargin (Tg) (30 nM) or left untreated, as control for 48 h. Data are expressed relative to the geometric mean of the starting concentration of reference genes (GAPDH and B2M). The histograms represent the mRNA expression levels of BRCA-1 genes of three different experiments. Data are represented as the mean relative to the control plus S.D. *p value < 0.05. **B** RKO were treated with Thapsigargin (10 or 30 nM) or left untreated as control. After 48 h the expression of BRCA-1, CHOP, γH2AX, XBP1s was evaluated by western blot analysis. β-Actin was used as loading control. The histograms represent the mean plus S.D. of the densitometric analysis of the ratio between the protein and β-Actin. *p < 0.05. **C** qRT-PCR of BRCA-1 in RKO treated with Thapsigargin (10 or 30 nM) or left untreated as control for 48 h. Data are expressed relative to the geometric mean of the starting concentration of reference genes (GAPDH and B2M). The histograms represent the mRNA expression levels of BRCA-1 genes of three different experiments. Data are represented as the mean relative to the control plus S.D. *p value < 0.05. **D** RKO cells were pre-treated or not with ceapinA7 (12 μM) for 1 h and then treated or not with Thapsigargin (10 nM) or left untreated, as control. After 48 h of treatments, the expression of BRCA-1, γH2AX, and CHOP was evaluated by Western blot analysis. β-Actin was used as loading control. The histograms represent the densitometric analysis of the ratio of specific protein/β-Actin of three different experiments. Data are represented as the mean plus S.D. *p value < 0.05. **E** RKO cells were pre-treated or not with 4u8c (10 μM) for 1 h and then treated or not with Thapsigargin (30 nM) or left untreated, as control. After 48 h of treatments, the expression of BRCA-1, γH2AX, CHOP, and XBP1s was evaluated by western blot analysis. β-Actin was used as loading control. The histograms represent the densitometric analysis of the ratio of specific protein/β-Actin of three different experiments. Data are represented as the mean plus S.D. *p value < 0.05.

To evaluate whether BRCA-1 downregulation could correlate with a reduced mRNA expression, we performed a qRT-PCR in cells treated by DPE, Thapsigargin or by the combination of these ER stressors with ceapinA7. As shown in Fig. 3A, Thapsigargin reduced BRCA-1 mRNA expression while DPE did not, mirroring the results obtained at protein level. Interestingly, the combination of Thapsigargin or DPE with ceapinA7 did not influence BRCA-1 mRNA expression compared to the single treatments (Fig. 3A), suggesting that downregulation of BRCA-1 observed following DPE/ceapinA7 or Thapsigargin/ceapinA7 combination treatments occurred at post-transcriptional level. Of note, the downregulation of BRCA-1 mediated by Thapsigargin as single treatment, occurring at 30 nM, was very slight at 10 nM, either at protein (Fig. 3B) and mRNA level (Fig. 3C) suggesting that it was a dose-dependent effect. Indeed, at 30 nM dose, Thapsigargin induced a stronger DNA damage and ER stress, as demonstrated by stronger the upregulation of γH2AX, CHOP, and XBP1s (Fig. 3B). However, ATF6 inhibition by ceapinA7 still reduced BRCA-1 expression level and increased γH2AX and CHOP expression level also in cancer cells treated by Thapsigargin at low dose (10 nM) (Fig. 3D).

Besides XBP1, the endoribonuclease activity of IRE1 alpha may affect several mRNAs, activity known as RIDD [18]. Therefore, having observed a reduced BRCA-1 mRNA expression and a stronger ER stress in colon cancer cells treated by Thapsigargin at the dose of 30 nM, we investigated whether BRCA-1 downregulation could depend on the IRE1 alpha endoribonuclease activity. To address this question, we inhibited it by 4μ8c and observed that this drug restored BRCA-1 expression and concomitantly counteracted the upregulation of γH2AX induced by Thapsigargin (Fig. 3E). All together these results suggest that Thapsigargin at 30 nM dose activated the RIDD activity of IRE1 alpha that was responsible for the reduction of BRAC-1 mRNA. These results also suggest that the combination of ceapinA7 with DPE or Thapsigargin reduced BRCA-1 expression at post-transcriptional level.

### ATF6 silencing mirrors the effect of ceapinA7 in reducing BRCA-1 expression in DPE- and Thapsigargin-treated colon cancer cells

**Fig. 4:**
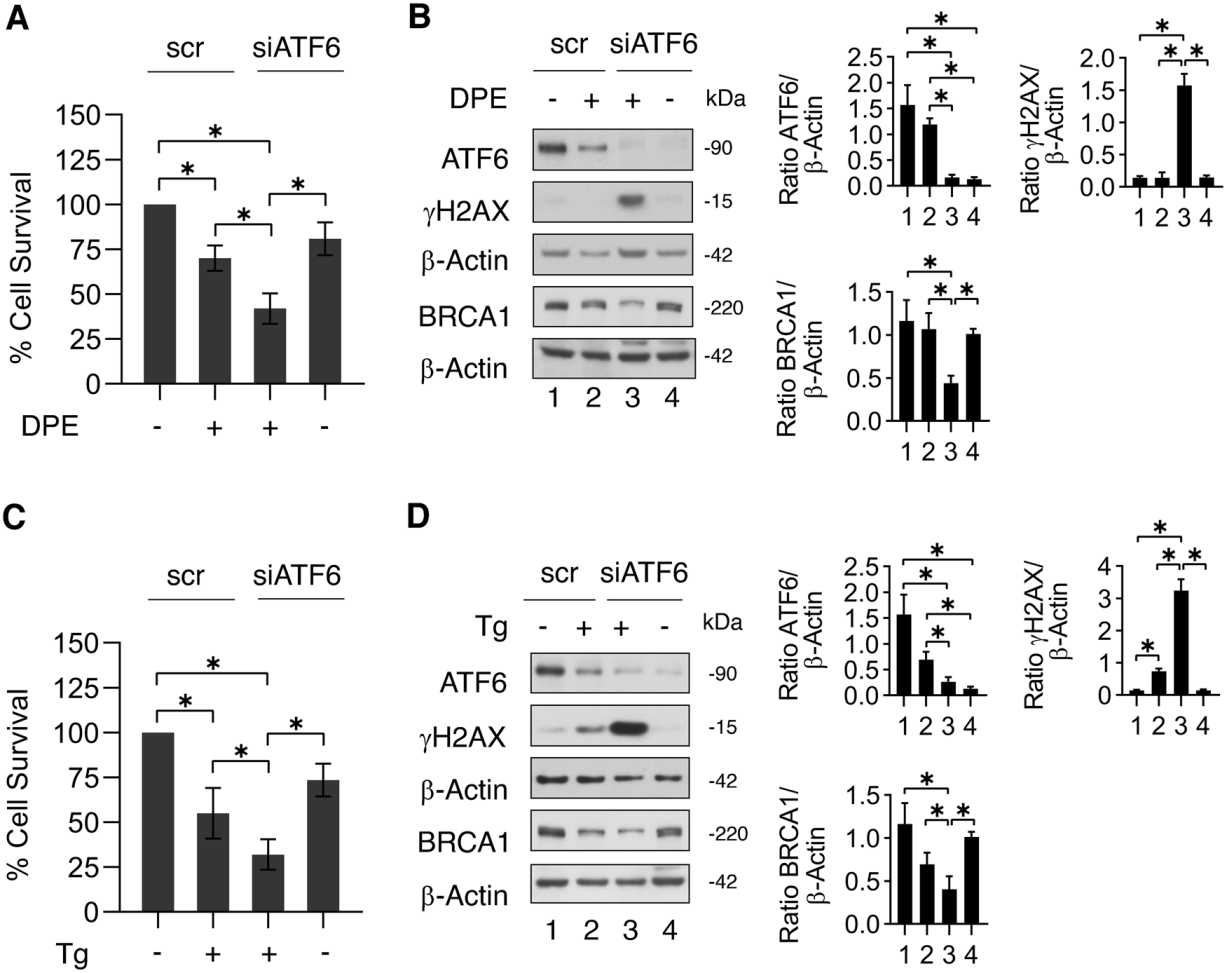
ATF6 silencing confirms that ATF6 sustains BRCA-1 expression and protects against DNA damage cancer cells subjected to ER stress. ATF6 was silenced in RKO cells and after 24 h cells were treated or not with DPE (100 μM) for 48 h. **A** Cell viability was measured by a Trypan Blue exclusion assay; the histograms represent the mean ± S.D. of live cells as percent of scramble control (scr) cells. *p-value < 0.05. **B** Protein expression level of ATF6, γH2AX, and BRCA-1 was evaluated by western blot analysis. β-Actin was used as loading control. The histograms represent the densitometric analysis of the ratio of specific protein/β-Actin of three different experiments. Data are represented as the mean plus S.D. *p-value **<** 0.05. ATF6 was silenced in RKO cells and after 24 h cells were treated or not with Thapsigargin (Tg) (30 nM) for 48 h. **C** Cell viability was measured by a Trypan Blue exclusion assay; the histograms represent the mean ± S.D. of live cells as percent of scramble control (scr) cells. *p-value < 0.05. **D** Protein expression level of ATF6, γH2AX, and BRCA-1 was evaluated by western blot analysis. β-Actin was used as loading control. The histograms represent the densitometric analysis of the ratio of specific protein/β-Actin of three different experiments. Data are represented as the mean plus S.D. *p-value < 0.05.

To further demonstrate the role of ATF6 in protecting stressed cancer cells from cell death, preventing DNA damage, and sustaining the expression of BRCA-1, we silenced ATF6 prior exposing cells to DPE or Thapsigargin. As shown in Fig. 4A, C, cell survival was reduced in ATF6 silenced cells in comparison to those scramble-treated and exposed to both ER stressors.

Accordingly, ATF6 silencing induced a stronger DNA damage in terms of γH2AX expression level and further downregulated BRCA-1 in cancer cells stressed by DPE or Thapsigargin (Fig. 4B, D). These effects mirrored those obtained by pharmacological inhibition of ATF6 by ceapinA7 and confirm the role of ATF6 in protecting cells from DNA damage induced by these ER stressors.

### ATF6 sustains mTOR activation and HSP90 expression, stabilizing BRCA-1 and preventing its proteasomal degradation

**Fig. 5:**
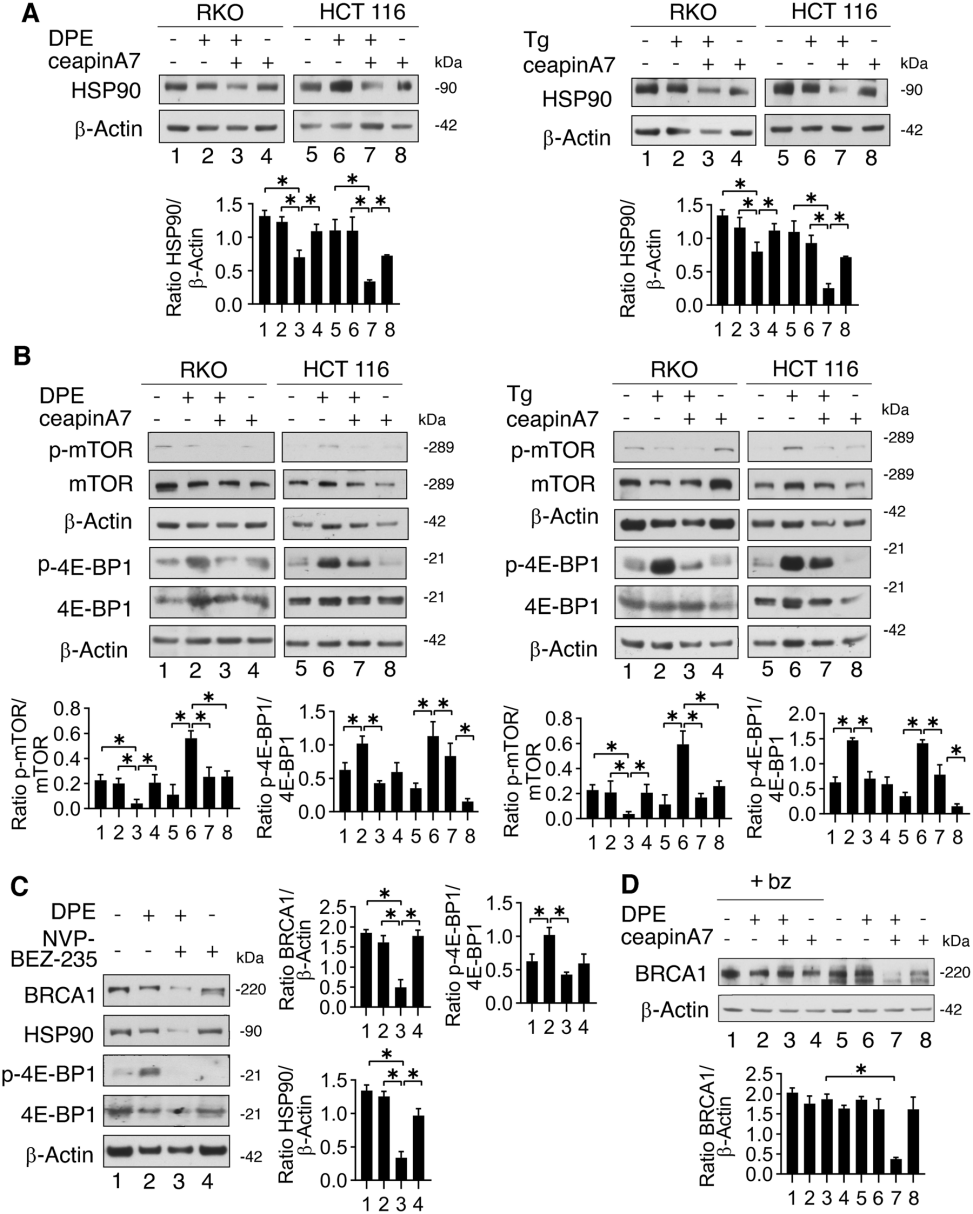
ATF6 sustains mTOR activation and HSP90 expression during ER stress which stabilizes BRCA-1 and prevents its proteasomal degradation. RKO and HCT116 cells were pre-treated or not with ceapinA7 (12 μM) for 1 h and then treated or not with DPE (100 μM) or Thapsigargin (Tg) (30 nM) or left untreated, as control. **A**, **B** Protein expression level of HSP90, p-mTOR, mTOR, p-4EBP1, and 4EBP1 was evaluated by western blot analysis. β-Actin was used as loading control. The histograms represent the densitometric analysis of the ratio of specific protein and the appropriate control of three different experiments. Data are represented as the mean plus S.D. *p-value < 0.05. **C** RKO cells were pre-treated or not with NVP-BEZ-235 (250 nM) for 1 h and then treated or not with DPE (100 μM) or left untreated, as control. Protein expression level of BRCA-1, HSP90, p-4EBP1, and 4EBP1 was evaluated by western blot analysis. β-Actin was used as loading control. The histograms represent the densitometric analysis of the ratio of specific protein and the appropriate control of three different experiments. Data are represented as the mean plus S.D. *p-value < 0.05. **D** RKO cells were pre-treated or not with ceapinA7 (12 μM) for 1 h and then treated or not with DPE (100 μM) for 48 h. To evaluate proteasomal degradation cells were incubated or not with Bortezomib (bz) (10 nM) for the last 4 h of treatments. Western blot analysis of BRCA-1 expression level. β-Actin was used as loading control and one representative experiment is shown. The histograms represent the densitometric analysis of the ratio of BRCA-1/β-Actin of three different experiments. Data are represented as the mean plus S.D. *p* value: *< 0.05.

After having demonstrated that BRCA-1 downregulation by ceapinA7 in combination with DPE or Thapsigargin was not due to a reduced mRNA expression and considering that BRCA-1 stability is strongly dependent on the expression of HSP90, being a client protein of this chaperone [19], we investigated the expression of HSP90 in cancer cells undergoing DPE or Thapsigargin treatments with or without ceapinA7. As shown in Fig. 5A, HSP90 was downregulated by the DPE/ceapinA7 or Thapsigargin/ceapinA7 combination treatments both in RKO and HCT116 cells compared to the single treatments. ATF6 has been reported to activate mTOR in the course of ER stress [13, 15], and a cross-talk between mTOR and HSP90 has been previously demonstrated [15, 20]. Therefore, we then investigated mTOR activation and evaluated the phosphorylation of its target p-4EBP1 and found that both were reduced in cells treated by ceapinA7 in combination with DPE or Thapsigargin (Fig. 5B). The role of mTOR in sustaining HSP90 and BRCA-1 expression level in stressed cancer cells was demonstrated in this setting by using NVB-BEZ-235, dual mTOR inhibitor that strongly downregulated both proteins (Fig. 5C). All together these results suggest that ATF6 was sustaining HSP90 and BRCA-1 expression level by triggering mTOR activation. Finally, to assess whether, due to an impairment of its stability, BRCA-1 could undergo to an increased proteasomal degradation following ATF6 inhibition, we used the proteasome inhibitor Bortezomib (bz). As shown in Fig. 5D, the expression of BRCA-1 was restored by bz in stressed cells, suggesting that ATF6 inhibition promoted its degradation via proteasome.

### ATF6 inhibition potentiates the cytotoxic effect of Adriamycin in DPE- or Thapsigargin-treated cells

**Fig. 6:**
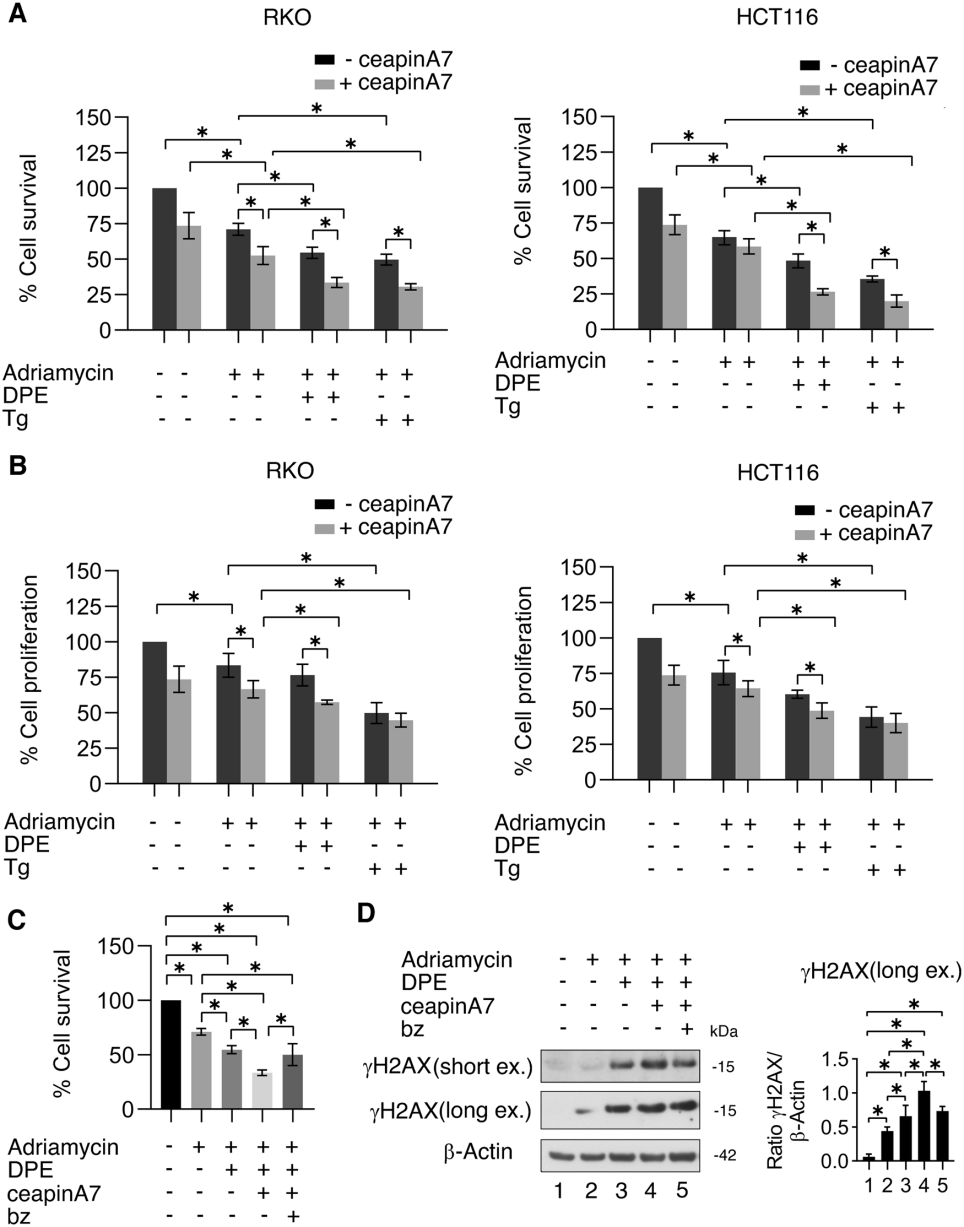
ATF6 inhibition enhances Adriamycin-mediated reduction of cell survival and cell proliferation in cells treated with DPE or Thapsigargin. RKO and HCT116 cells were pre-treated or not with ceapinA7 (12 μM) for 1 h and then treated or not with DPE (100 μM) or Thapsigargin (Tg) (30 nM) or left untreated, as control. After 3 h of culture, Adriamycin (1 μg/ml) was added to the cultures. **A** Cell viability was measured by a Trypan Blue exclusion assay after 48 h of culture, the histograms represent the mean ± S.D. of live cells as percent of untreated control cells. *p* value: *< 0.05. By using the KERN index (R), we found that ceapinA7 induced a synergistic cytotoxic effect in combination with DPE/Adriamycin in both RKO and HCT116 (R > 1) and in combination with Thapsigargin/Adriamicin in HCT116 cells (R > 1) while in RKO cells ceapinA7 combined with Thapsigargin/Adriamicin induced an additive effect (R = 1). **B** Cell proliferation was measured by MTT assay after 48 h of culture, the histograms represent the mean ± S.D. of the ratio between the OD of treated cells and control cells. *p* value: *< 0.05. **C** RKO cells were pre-treated or not with ceapinA7 (12 μM) for 1 h and then treated or not with DPE (100 μM). After 3 h of culture, Adriamycin (1 μg/ml) was added to the cultures. Proteasome inhibitor bortezomib (bz) was added to the cultures for the last 6 h of treatments. Untread cells were used as control. Cell viability was measured by a Trypan Blue exclusion assay after 48 h of culture, the histograms represent the mean ± S.D. of live cells as percent of untreated control cells. *p* value: *< 0.05. **D** Protein expression level of γH2AX was evaluated by western blot analysis. β-Actin was used as loading control. The histograms represent the densitometric analysis of the ratio of γH2AX/β-Actin of three different experiments. Data are represented as the mean plus S.D. *p-value < 0.05.

We then evaluated whether the reduction of BRCA-1 by DPE/ceapinA7- or Thapsigargin/ceapinA7 could potentiate the cytotoxic effect of a DNA damaging agent such as Adriamycin, widely used to treat colon cancer [21]. We found that DPE and Thapsigargin, as single agents, increased the cytotoxicity of Adriamycin and that the cytotoxic effect further increased when cells were treated with DPE or Thapsigargin in combination with ceapinA7 (Fig. 6A). Cell proliferation was also further reduced by Adriamycin in cells treated by DPE or Thapsigargin in combination with ceapinA7 (Fig. 6B). These results suggest that the reduction of BRCA-1 expression induced by DPE/ceapinA7 or Thapsigargin/ceapinA7 combinations rendered colon cancer cells more sensitive the cytotoxic effect of Adriamycin. We next evaluated whether bz that prevented BRCA-1 degradation induced by DPE/ceapinA7 could reduce the cytotoxic effect of these drugs in combination with Adriamycin. As shown in Fig. 6C we found that bz reduced the cytotoxicity of DPE/ceapinA7/Adriamycin treatment and, accordingly, the expression of γH2AX was lower in the presence of bz in DPE/ceapinA7/Adriamycin-treated cells (Fig. 6D).

### CeapinA7 downregulates BRCA-1, increases DNA damage, and potentiates the cytotoxic effect of Adriamycin also in HCT116 p53−/− cells undergoing ER stress

**Fig. 7:**
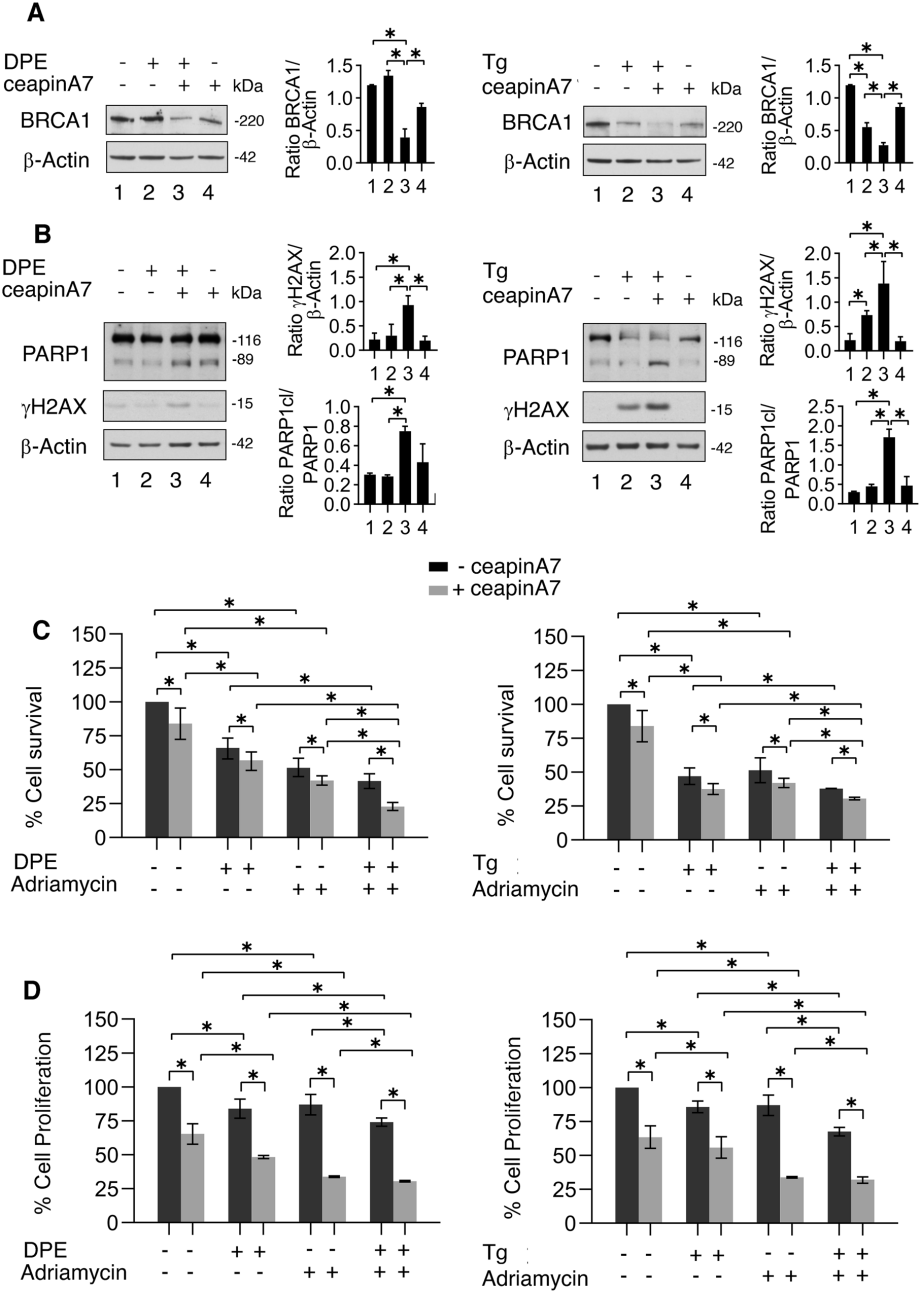
CeapinA7 reduces BRCA-1, increases DNA damage, and the sensitivity to Adriamycin also in HCT116 p53−/− cells treated by DPE or Thapsigargin. HCT116 p53−/− cells were pre-treated or not with ceapinA7 (12 μM) for 1 h and then treated or not with DPE (100 μM) or Thapsigargin (Tg) (30 nM) or left untreated, as control, for 48 h. **A**, **B** Western blot analysis showing the expression level of BRCA-1, PARP cleavage and γH2AX. β-Actin was used as loading control and one representative experiment is shown. The histograms represent the densitometric analysis of the ratio of specific protein/β-Actin of three different experiments. Data are represented as the mean plus S.D. *p* value: *< 0.05. HCT116 p53−/− cells were pre-treated or not with ceapinA7 (12 μM) for 1 h and then treated or not with DPE (100 μM) or Thapsigargin (Tg) (30 nM) or left untreated, as control. After 3 h of culture, Adriamycin (1 μg/ml) was added or not to the cultures. **C** Cell viability was measured by a Trypan Blue exclusion assay after 48 h of culture, the histograms represent the mean ± S.D. of live cells as percent of untreated control cells. *p* value: *< 0.05. By using the KERN index (R), we found that ceapinA7 induced a synergistic cytotoxic effect when combined with Thapsigargin, Adriamycin and DPE/Adriamycin (R > 1) while in combination with DPE and Thapsigargin/Adriamycin the effect of ceapinA7 was additive (R = 1). **D** Cell proliferation was measured by MTT assay after 48 h of culture, the histograms represent the mean ± S.D. of the ratio between the OD of treated cells and control cells. *p* value: *< 0.05.

p53 activation has been reported to contribute to BRCA-1 downregulation [22, 23], therefore to evaluate the role of p53 we repeated the experiments in HCT116 p53−/− cells, exposing them with DPE or Thapsigargin in the presence or in the absence of ceapinA7. As shown in Fig. 7A, BRCA-1 downregulation occurred also in HCT116 p53−/− cells following DPE/ceapinA7 combination treatment and accordingly PARP cleavage and γH2AX expression increased in these cells (Fig. 7B). We then found that cell survival was further reduced by Adriamycin also in HCT116 p53−/− cells treated by DPE/ceapinA7 or Thapsigargin/ceapinA7 combination (Fig. 7C) and that cell proliferation was also impaired (Fig. 7D). These results suggest that the ATF6 prevents DNA damage and cell death also in stressed colon cancer cells carrying dysfunctional wtp53.

## Discussion

UPR targeting has been identified as a promising approach to treat cancers, including colon cancer [11]. Interestingly, UPR is triggered concomitantly with DDR in response to conditions of stress such as such as hypoxia and both responses may limit, at least to some extent, cancer cell death [24]. It has been shown that UPR and DDR share several target molecules and that the activation of UPR sensors may influence the execution of DNA damage repair [4]. In particular, UPR activation may lead to an impairment of DDR and increase the susceptibility of cancers to the treatments by DNA damaging agents, as reported for example in glioblastoma [25]. At this regard, we have recently reported that ER stress/UPR activation induced by Zinc chloride triggered a stronger p53 activation and upregulated CHOP in lymphoma cells exposed to low dose radiation, potentiating their-mediated cytotoxic effect [10].

**Fig. 8:**
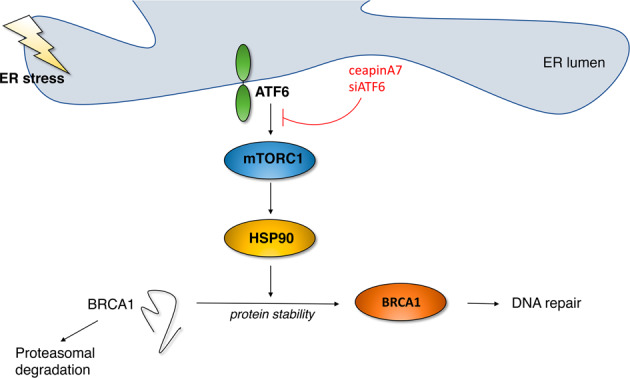
Representative scheme illustrating the role of ATF6 in sustaining the mTOR/HSP90/BRCA1 axis. ATF6 pharmacological or genetic inhibition reduces BRCA-1 stability in stressed cells and promotes its proteasomal degradation.

Although how the single UPR arm activation may influence DDR remains to be fully elucidated, it is known that IRE1 alpha/XBP1s axis can regulate the expression of several DDR molecules, either at transcriptional level, through the transcription factor XBP1s, or by mediating mRNA degradation, through IRE1 alpha-dependent decay (RIDD) [4]. Interestingly, the inhibition of IRE1 alpha endoribonuclease has been reported to potentiate the anti-cancer effects of genotoxic treatments, either in vitro and in vivo [26, 27]. In correlation with the RIDD activity of IRE1 alpha, in this study, we found that Thapsigargin, used at a 30 nM dose, induced a stronger ER stress and activation of XBP1s, reducing BRCA-1 mRNA expression. Indeed, BRCA-1 downregulation was counteracted by the IRE1 alpha endoribonuclease inhibitor 4μ8c, that also prevented DNA damage in Thapsigargin-treated colon cancer cells. However, the new finding of this study is that it shows for the first time that ATF6 arm of UPR plays a key role in DNA repair, by sustaining BRCA-1 expression in colon cancer cells undergoing ER stress. This effect occurred in the course of mild as well as strong ER stress, induced by DPE or Thapsigargin. At molecular level, ATF6 activated mTOR, in agreement with previous studies [13, 28] and this pathway in turn sustained the expression level of HSP90 and the stabilization of BRCA-1 (Fig. 8), as reported for this and other DDR proteins [29]. The results of this study are also in agreement with the role of mTOR in positively regulating the expression of HSPs, previously observed by ours and other’s laboratories [15, 30]. Both ATF6 pharmacological and genetic inhibition resulted in mTOR inhibition, HSP90 downregulation and reduced BRCA-1 stability, promoting its proteasomal degradation. These findings suggest that the inhibition of ATF6, besides potentiating the cytotoxic effect of ER stressors against colon cancer cells, could be exploited to potentiate the anti-cancer effect of DNA damaging agents, due to the impairment of DDR. Interestingly, we have also shown that ATF6 inhibition sensitized stressed cancer cells to the cytotoxic effect of Adriamycin, a drug widely employed for the treatment of colon cancer. Exacerbating ER stress or manipulating UPR, besides leading to an unbalance of UPR towards cell death may therefore alter DDR and potentiate the cytotoxic effect of DNA damaging agents. Of note, if it is possible to interfere with DDR by manipulating UPR sensors, it is also possible that the induction of DNA damage may activate UPR, as reported for example in the case of IRE1α-dependent RNA decay activation by genotoxic stress [31].

Future studies will better elucidate whether the use of small molecules inhibiting the proteins in charge for DNA repair could unbalance UPR and affect its-mediated cell death/survival decision. In conclusion, this study sustains the new emerging findings suggesting that the manipulation of UPR, DDR, and their interplay may transform these adaptive responses into lethal weapons against cancer.

## Materials and methods

### Cell cultures and treatments

RKO (human colon cancer cell line, wtp53), HCT116 (human colon cancer cell line, wtp53), and HCT116 p53−/− (human colon cancer cell line, p53 K/O) were a kind gift from B. Vogelstein (Johns Hopkins University, Baltimore, MD). Cells were maintained in DMEM (PAN-Biotech, Aidenbach, Germania) supplemented with 10% Fetal Bovine Serum (FBS) (Corning, Corning, NY, USA), L-glutamine (100 μg/ml) (Aurogene, Rome, Italy), streptomycin and penicillin (100 U/ml) (Aurogene) at 37 °C in a 5% CO_2_ incubator. Cells were always detached using Trypsin-EDTA solution (Aurogene). Cells were plated in 12-well plates at a density of 10^5^ cells/well in 1 ml and treated the following day. The sensitivity of the RKO and HCT116 cells to DPE was previously evaluated [12], while to analyze Thapsigargin (Tg) (Sigma-Aldrich, Burlington, MA, USA) sensitivity, cells were treated with subsequent concentrations of Thapsigargin (range between 10 and 200 nM) for 48 h. In some experiments cells were pre-treated for 1 h with 12 μM ceapinA7 (Sigma-Aldrich), ATF6 signaling blocker, or 10 μM 4μ8C (Sigma-Aldrich), selective IRE1 Rnase inhibitor, or 250 nM NVP-BEZ-235 (Selleckchem, Houston, TX, USA) dual PI3K/mTOR inhibitor, and then treated with 100 μM DPE (Cayman Chemical Company, Ann Arbor, MI, USA) or 10 or 30 nM Thapsigargin for 48 h. To evaluate proteasomal degradation cells were incubated or not with 10 nM Bortezomib (BZ) (Sigma-Aldrich) for the last 4 or 6 h of treatments. In some experiments, 1 μg/ml Adriamycin (Sigma-Aldrich) was added to the cultures 3 h after the first treatment. Untreated cells were used as control.

### ATF6 silencing

ATF6 knockdown was performed by specific siRNA transfection into RKO cells. Briefly, cells were plated in 12-well plates at a density of 5 × 10^4^ cells/well in 1 ml and, the day after, when cell were 30% confluency 1.8 pmol of siRNA were transfected using INTERFERin transfection reagent (Polypolus Transfection, Illkirch-Graffenstaden, France) according to the manufacturer’s instructions. Control siRNA-A (Santa Cruz Biotechnology, Dallas, TX, USA) was used as a scrambled control (scr). After 24 h, cells were treated with 100 μM DPE or 30 nM Thapsigargin for additional 48 h.

### Cell viability

Cell viability was evaluated by a Trypan Blue (Sigma-Aldrich) exclusion assay after 24 or 48 h of culture. Cells were counted by light microscopy using a Neubauer hemocytometer. The experiments were performed in triplicate and repeated at last three times.

### MTT assay

RKO, HCT116, and HCT116 p53−/− cells were plated in 96-well plates at the density of 5 × 10^3^ cells/well in 100 μL of complete medium. The day after, cells were treated as above described for 48 h. After treatments, cells were washed once with PBS and then MTT assay (Sigma-Aldrich) was performed following manufacturer’s instruction. The intensity of formazan staining was measured at 560 nm by Absorbance 96 reader (Byonoy GmbH, Hamburg, Germany). The experiments were performed in triplicate and repeated three times.

### Western blot analysis

The preparation of whole-cell protein lysates and the procedures for western blotting were the same as previously described [32]. All original blots are included as supplementary materials. To evaluate protein expression on western blot membranes the primary antibody used were mouse monoclonal anti-p53 (clone DO-1) (sc-126, Santa Cruz Biotechnology), rabbit monoclonal anti-PARP (46D11) (9532; Cell Signaling, Danvers, MA, USA) rabbit polyclonal anti-BiP/GRP78 (11587-1-AP; Proteintech, Rosemont, IL, USA), rabbit monoclonal anti-CHOP (66741-1-Ig; Proteintech), mouse monoclonal anti-γH2AX (Ser 139) (sc-517348; Santa Cruz Biotechnology), mouse monoclonal anti-BRCA-1 (OP92; EMD Millipore, Burlington, MA, USA), rabbit polyclonal anti-CHOP (GADD153) (15204-1-AP; Proteintech), rabbit polyclonal anti-XBP1s (24868-1-AP; Proteintech), rabbit polyclonal anti-ATF6 (24169-1-AP; Proteintech), rabbit polyclonal anti-HSP90 (13171-1-AP; Proteintech), rabbit polyclonal anti-p-mTOR (Ser2448) (2971; Cell Signaling), mouse monoclonal anti-mTOR (66888-1-Ig; Proteintech), rabbit monoclonal anti-Phospho-4E-BP1 (Thr37/46) (236B4; Cell Signaling), mouse monoclonal anti-4E-BP1 (60246-1-Ig; Proteintech). Mouse monoclonal anti-β-actin (A5316; Sigma-Aldrich) was used as loading control. The goat anti-mouse IgG-HRP (A90-116P; Bethyl Laboratories, Montgomery, TX, USA) and goat anti-rabbit IgG-HRP (A120-101P; Bethyl Laboratories) were used as secondary antibodies.

### Indirect immunofluorescence assay

Indirect immunofluorescence assay (IFA) was performed to evaluate γH2AX foci formation. RKO cells were grown on slides, pre-treated or not with ceapinA7 and then treated with DPE (100 μM) or Thapsigargin (30 nM). After 48 h of treatments, cells were processed as previously described [33]. Thereafter, cells were incubated with mouse primary monoclonal antibody anti-γH2AX (phosphor-Ser 139) (sc-517; Santa Cruz Biotechnology) for 1 h at room temperature. Subsequently, cells were incubated with a polyclonal conjugated-Cy3 sheep anti-mouse antibody (Jackson ImmunoResearch, Cambridge, UK) for 30 min at room temperature and stained with DAPI (Sigma-Aldrich) for 1 min at room temperature. Slides were analyzed with Apotome Axio Observer Z1 inverted microscope (Zeis, Wetzlar, Germany) equipped with an AxioCam MRM Rev.3 at ×40 magnification. Foci number analysis was performed by Image J software (1.47 version, NIH, Bethesda, MD, USA).

### RNA isolation and quantitative real-time polymerase chain reaction (qRT-PCR)

After treatments, total RNA from RKO was isolated with TRIzol™ Reagent (Invitrogen, Carlsbad, CA, USA) according to the manufacturer’s instructions. BRCA-1 mRNA expression levels were analyzed using TaqMan gene expression assays (Applied Biosystems, Vilniaus, Lithuania). Briefly, 2 µg of total RNA was reverse-transcribed into cDNA using High-capacity cDNA Reverse Transcription Kit (Thermo Fisher Scientific, Waltham, MA, USA). A mastermix containing 2 µL cDNA (20 ng), 1 µL of TaqMan gene expression assays specific for BRCA-1 (HS01556193-m1; Applied Biosystem), and 10 µL of 2x TaqMan Fast Advance Master Mix was prepared for each PCR. The PCRs were run on an Applied Biosystem Real-Time thermocycler. Each amplification was performed in triplicate, and the average of three threshold cycles was used to calculate transcript abundance. The starting concentration of each specific product was divided by the geometric mean of the starting concentration of reference genes (GAPDH and B2M) (HS99999905-m1 and HS99999907-m1; Applied Biosystem) and this ratio was compared between treated/control groups.

### Densitometric analysis

The quantification of protein bands was performed by densitometric analysis using the Image J software (1.47 version, NIH, Bethesda, MD, USA).

### Statistical analysis

Results are represented by the mean ± standard deviation (SD) of at least three independent experiments and a two-tailed Student’s *t*-test was used to demonstrate statistical significance. Difference was considered as statistically significant when *p*-value was at least <0.05.

## Supplementary information

Original Data File

## Data Availability

The datasets generated and/or analyzed during the current study are available from the corresponding author upon reasonable request.
