# Supplementary figures and images for: ATF6 prevents DNA damage and cell death in colon cancer cells undergoing ER stress
Source: Cell Death Discov. 2022 Jun 25;8:295. doi: 10.1038/s41420-022-01085-3 (PMC9233702; doi:10.1038/s41420-022-01085-3)

Fig. 1

1A

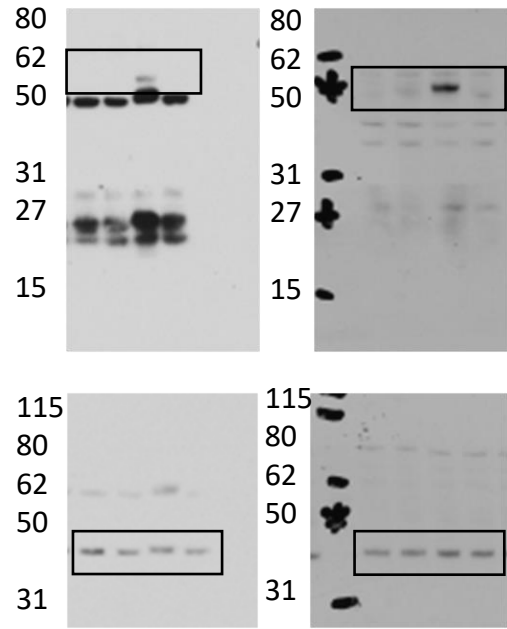

1B

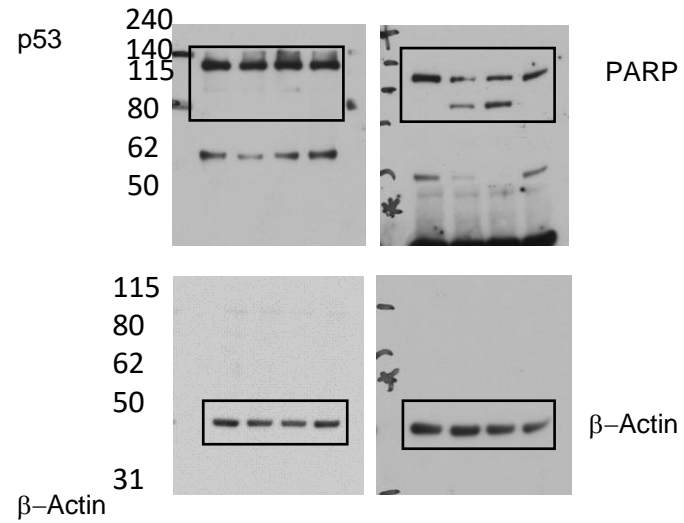

1C

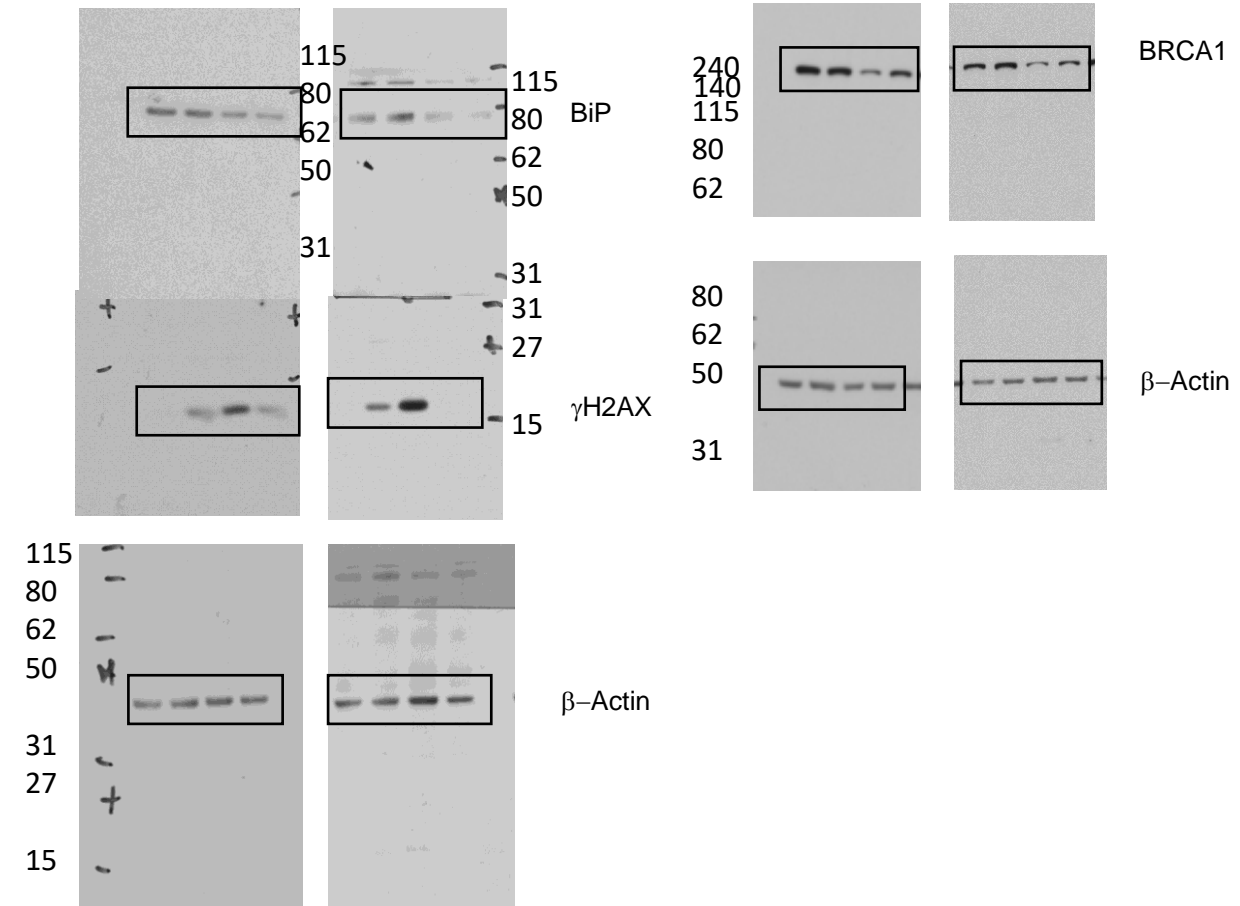

Fig. 2

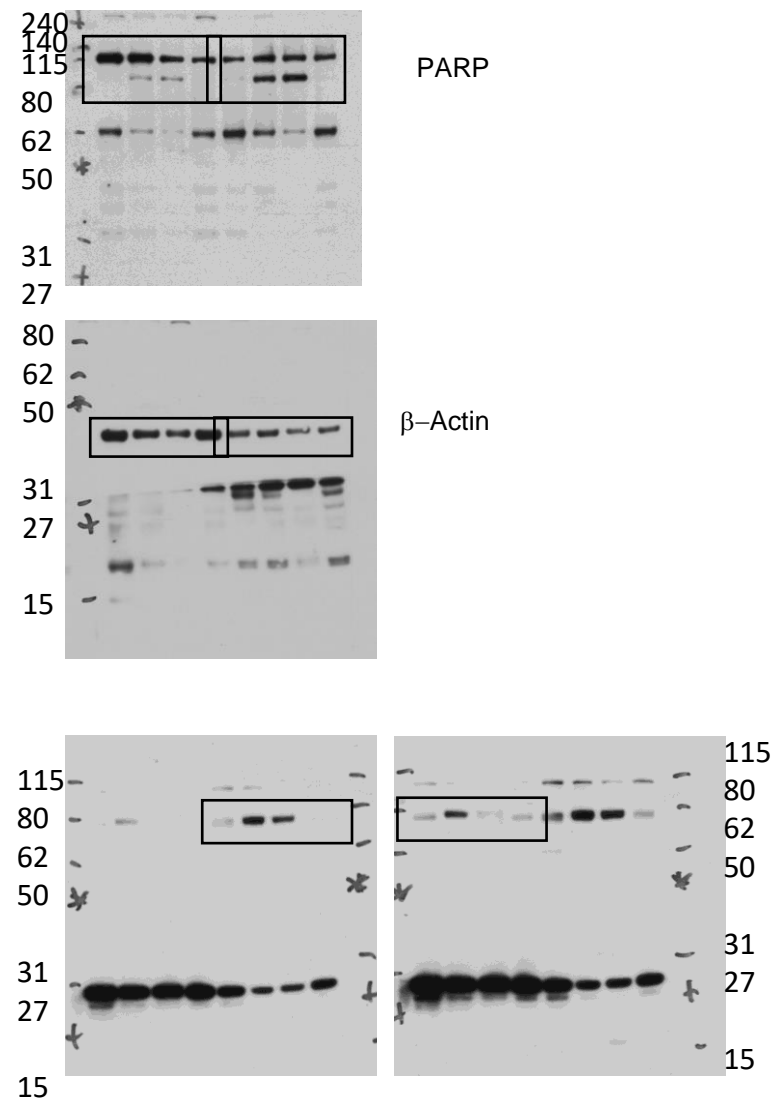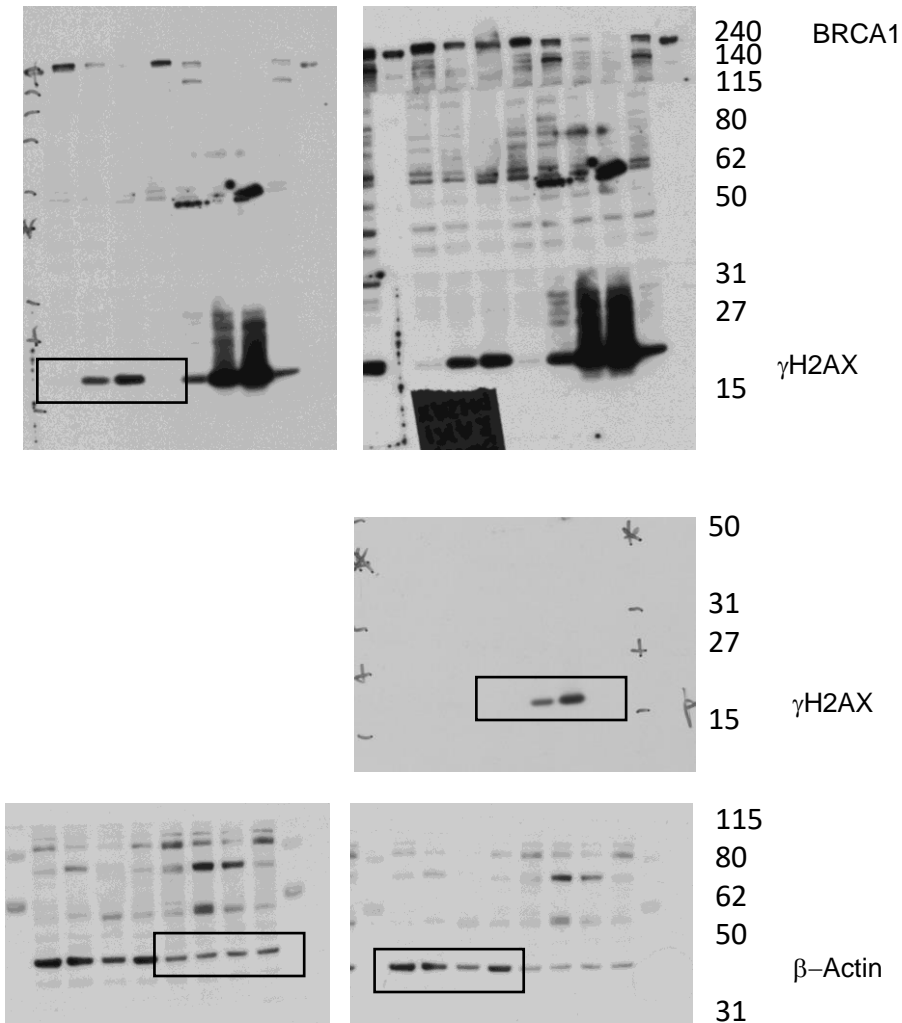

Fig. 3

3B

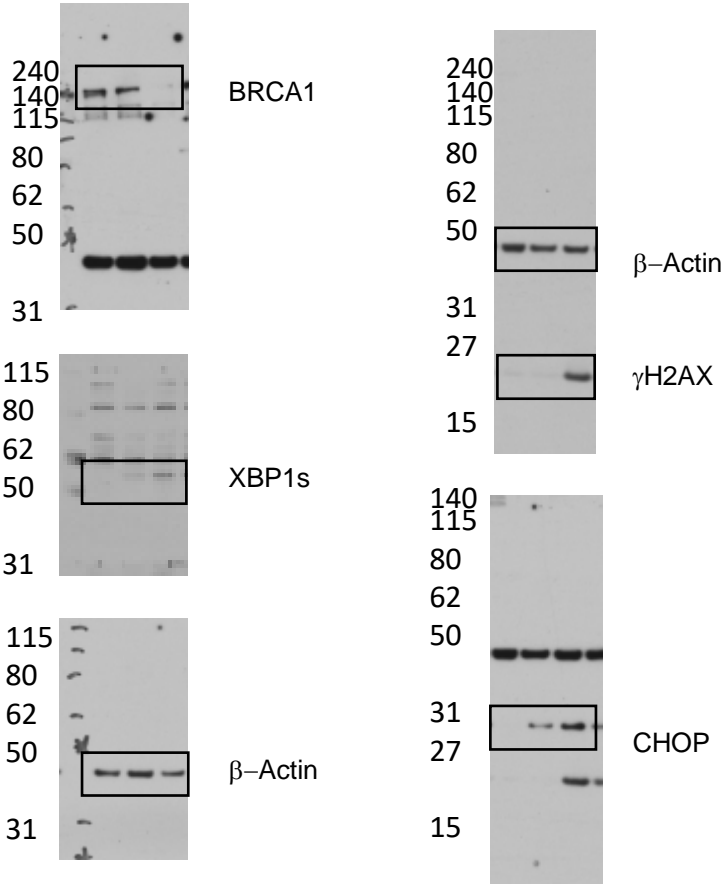

3D

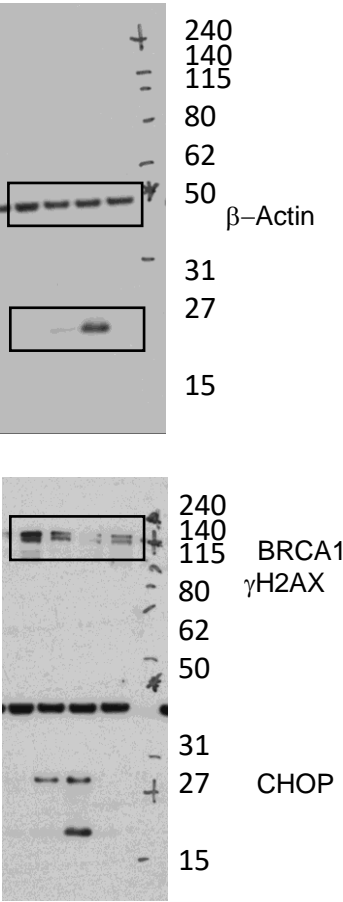

3E

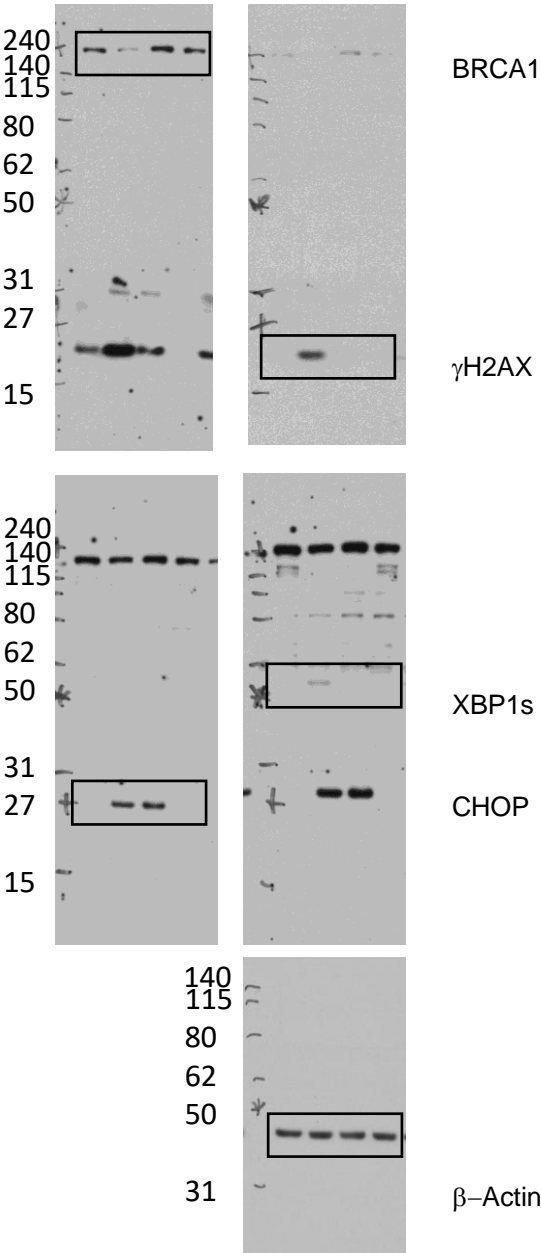

Fig.4

4B

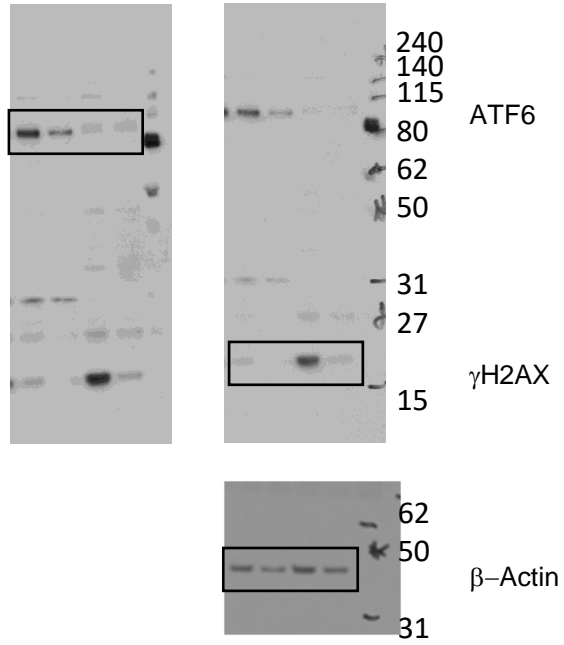

4D

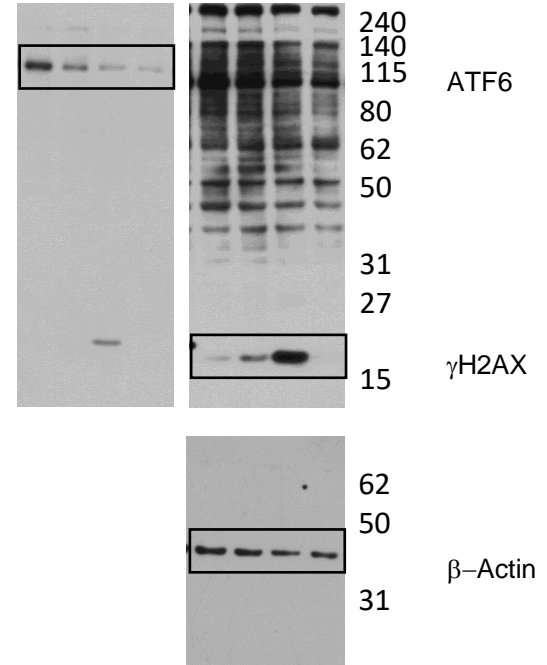

4B,D

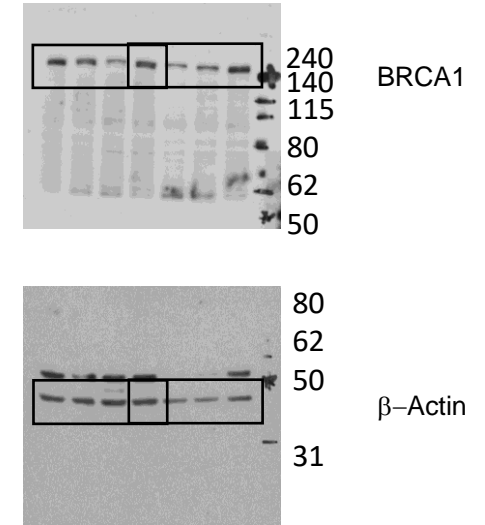

5A

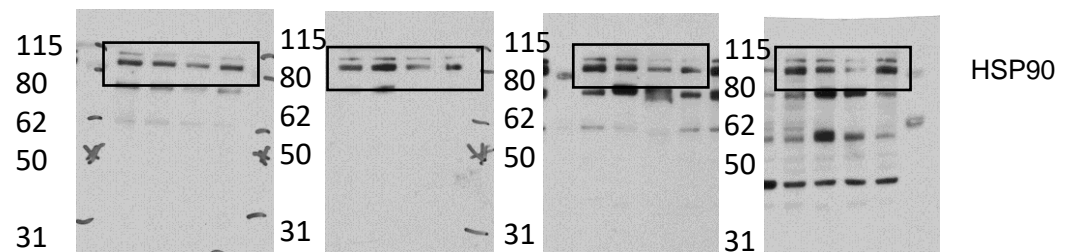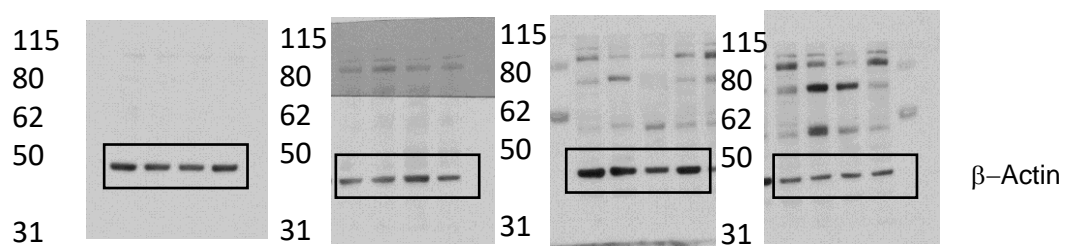

5B

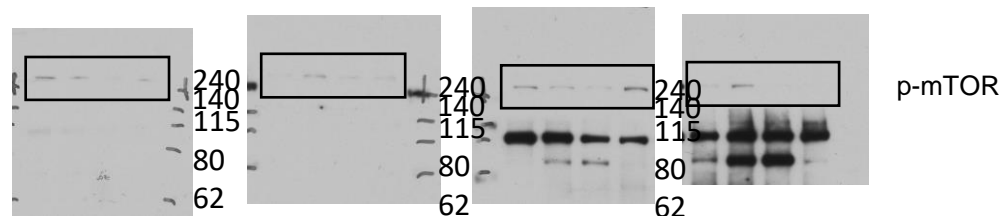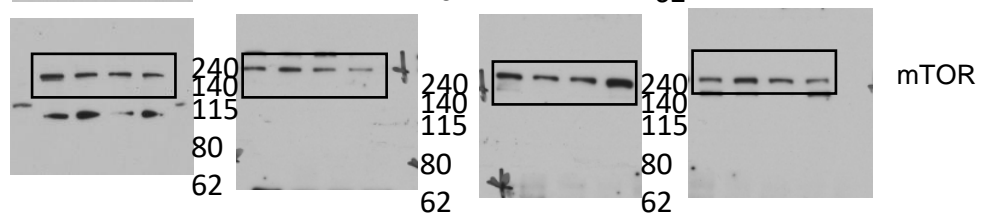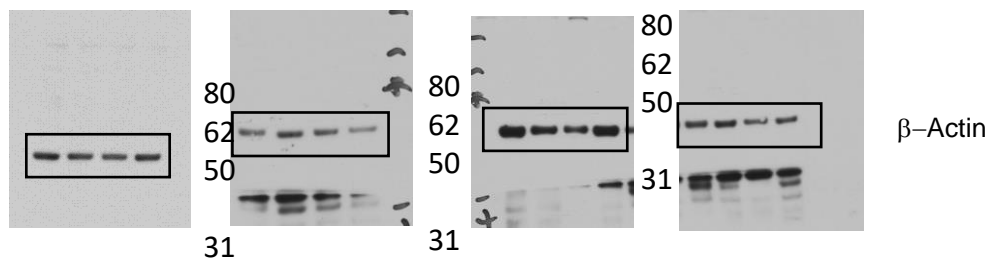

Fig.5

5B

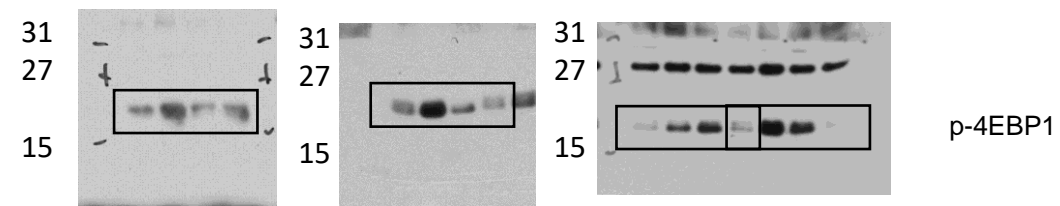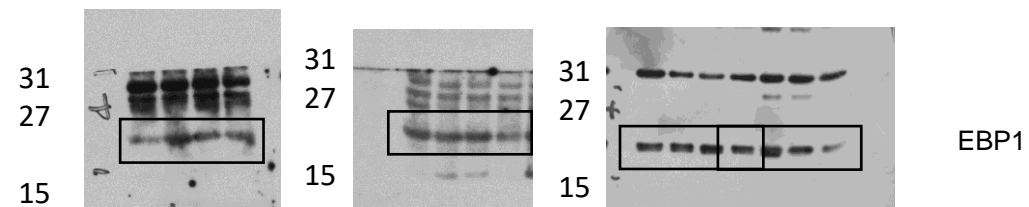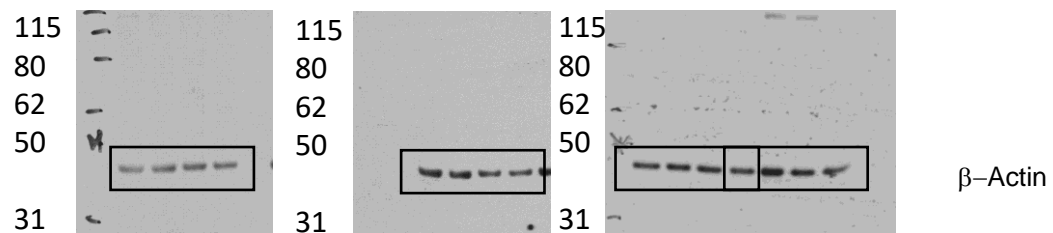

Fig.5

5C

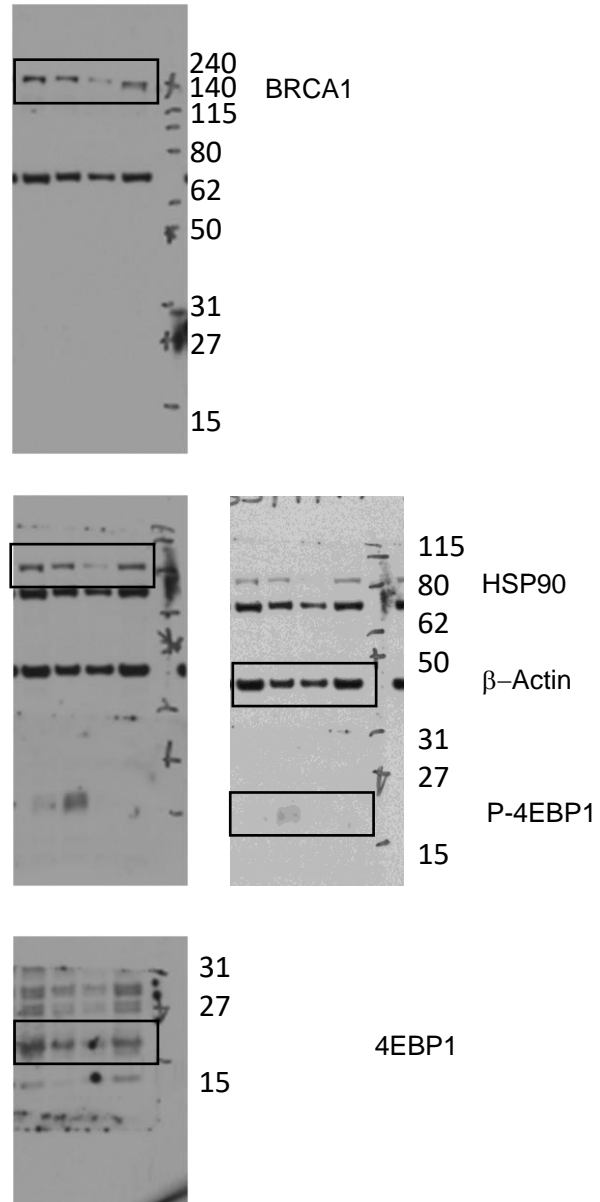

5D

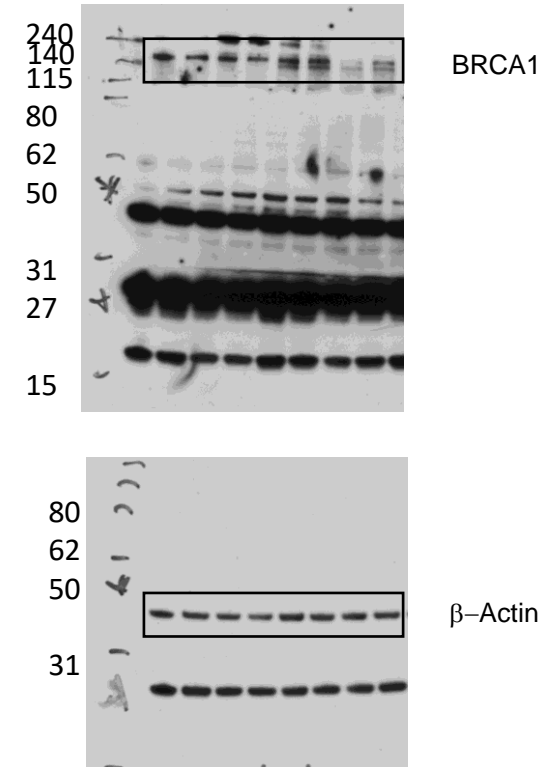

Fig.6

6D

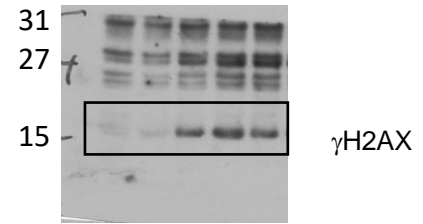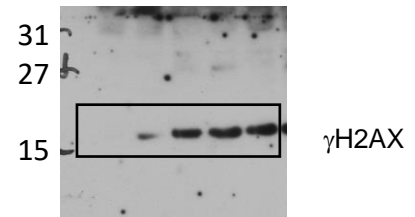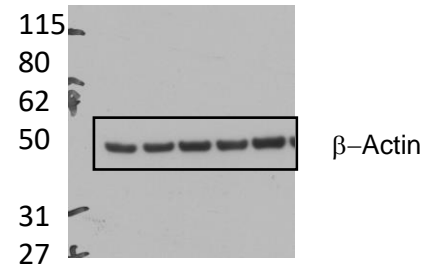

Fig.7

7A

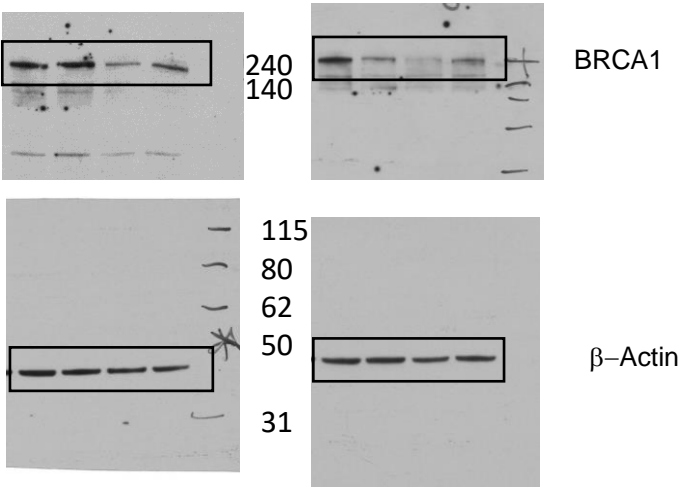

7B

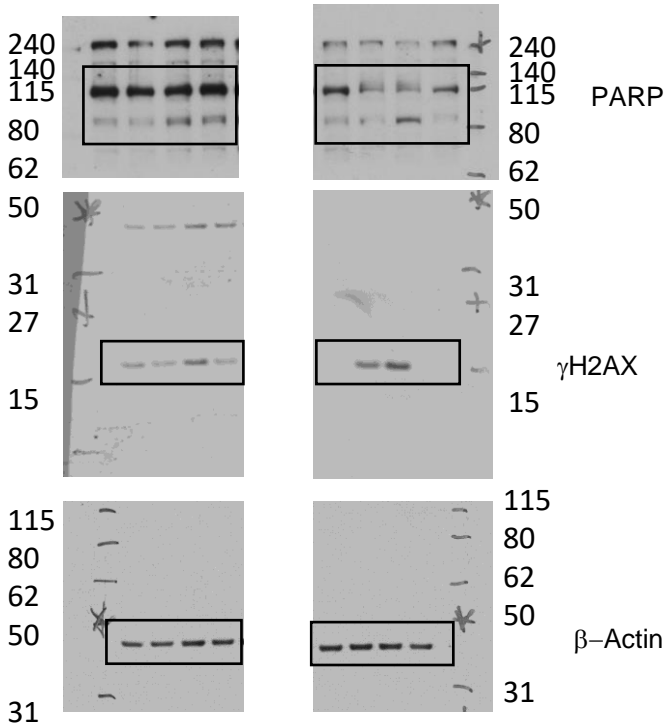

Supplement: Supplementary file 1 — Original Data File [file 41420_2022_1085_MOESM1_ESM.pdf]
